# Supplementary material for: Characterizing collaborative transcription regulation with a graph-based deep learning approach
Source: PLoS Comput Biol. 2022 Jun 6;18(6):e1010162. doi: 10.1371/journal.pcbi.1010162 (PMC9203014; doi:10.1371/journal.pcbi.1010162)
Supplement: S1 Table — (PDF) [file pcbi.1010162.s001.pdf]

Type	Notation	Explanation
Subscripts & superscripts	i	Index of a segment of DNA sequence, $i = 1, \ldots, N$
	l	Index of a chromatin feature, $l = 1, \ldots, L$
	c	Spatial relationship from a contact map
	s	Sequential relationship from DNA sequences
Operators	$\|$	Concatenation
	T	Transpose
	$\cup$	Union
Symbols	$\mathcal{V}$	Set of nodes
	$\mathcal{E}$	Set of edges
	$\mathcal{A}$	Weighted adjacency matrix
	$\mathcal{G}$	Graph: $\mathcal{G} = (\mathcal{V}, \mathcal{E}, \mathcal{A})$
	R	Set of real numbers
	$\mathcal{N}_s^{(i)}$	Sequential neighbor set of sequence i
	$\mathcal{N}_c^{(i)}$	Spatial neighbor set of sequence i
	$\mathcal{N}^{(i)}$	Neighbor set of sequence i: $\mathcal{N}^{(i)} = \mathcal{N}_s^{(i)} \cup \mathcal{N}_c^{(i)}$
Neural network layers & functions	f	Sequence layers
	g_c, g_s	Graph layers
	p	Prediction layer
	SAMPLE	Functions of sampling sequences from the neighborhood
	STACK	Functions of stacking multiple vectors to a matrix
Random variables	$x^{(i)}$	One-hot representation of 1000-bp DNA sequence
	$\hat{y}^{(i)}$	Predicted chromatin feature vector of sequence i
	$\hat{y}_l^{(i)}$	Predicted chromatin feature l on sequence i
	$\phi^{(i)}$	Hidden representation vector of sequence i extracted by sequence layers f
	$\Xi_s^{(i)}$	The feature matrix of sequence i as an input to graph layers g_s
	$\Xi_c^{(i)}$	The feature matrix of sequence i as an input to graph layers g_c
	$h_s^{(i)}$	Updated hidden representation of sequence i as an output from g_s
	$h_c^{(i)}$	Updated hidden representation of sequence i as an output from g_c
	$P_c^{(i)}$	Spatial sampling matrix
	$P_s^{(i)}$	Sequential sampling matrix
	$[S_c^{(i)}]_l$	Attribution scores of spatial sampling matrix $P_c^{(i)}$ for chromatin feature l
	$[S_s^{(i)}]_l$	Attribution scores of sequential sampling matrix $P_s^{(i)}$ for chromatin feature l
	$[V_c^{(i)}]_l$	Compressed interaction importance vector from $[S_c^{(i)}]_l$ for chromatin feature l
	$[V_s^{(i)}]_l$	Compressed interaction importance vector from $[S_s^{(i)}]_l$ for chromatin feature l
	M_l	Interaction importance matrix for chromatin feature l
	$[S^{(j)}]_l^{(i)}$	Attribution scores of sequence j for chromatin feature l
Parameters to be pre-specified	k_c	Number of sampled spatial neighbors
	k_s	Number of sampled sequential neighbors
	L	Number of chromatin features
	K	Length of hidden representation $\phi^{(i)}$
	N	Number of 1000-bp DNA sequence

S1 Table. Notations used in our work.
