# Supplementary material for: Characterizing collaborative transcription regulation with a graph-based deep learning approach
Source: PLoS Comput Biol. 2022 Jun 6;18(6):e1010162. doi: 10.1371/journal.pcbi.1010162 (PMC9203014; doi:10.1371/journal.pcbi.1010162)
Supplement: S2 Table — The first three models are the baselines, the fourth model is ECHO with only spatial neighbors sampled, and the fifth model is ECHO with only sequential neighbors sampled, and the last threes are our proposed methods built on the corresponding baseline model with 10 sequential neighbors and 50 spatial neighbors sampled. (PDF) [file pcbi.1010162.s002.pdf]

	Mean AUROC	Mean AUPR
DeepSEA	0.881	0.312
DanQ	0.881	0.316
DeepCNN	0.885	0.318
ECHO (built on DeepCNN with 50 spatial neighbors)	0.918	0.372
ECHO (built on DeepCNN with 100 sequential neighbors)	0.917	0.369
ECHO (built on DeepSEA)	0.918	0.373
ECHO (built on DanQ)	0.919	**0.386**
ECHO (built on DeepCNN)	**0.921**	0.378

S2 Table. Comparing the mean AUROC and AUPR scores of ECHO with the baselines. The first three models are the baselines, the fourth model is ECHO with only spatial neighbors sampled, and the fifth model is ECHO with only sequential neighbors sampled, and the last threes are our proposed methods built on the corresponding baseline model with 10 sequential neighbors and 50 spatial neighbors sampled.
