# Supplementary material for: Characterizing collaborative transcription regulation with a graph-based deep learning approach
Source: PLoS Comput Biol. 2022 Jun 6;18(6):e1010162. doi: 10.1371/journal.pcbi.1010162 (PMC9203014; doi:10.1371/journal.pcbi.1010162)
Supplement: S3 Table — (PDF) [file pcbi.1010162.s003.pdf]

	Mean AUROC	Mean AUPR	Mean Recall at 50% FDR
DeepCNN	0.900	0.377	0.330
ChromeGCN	0.916	0.406	0.372
ECHO	**0.924**	**0.429**	**0.399**

S3 Table. Comparing the performance of ECHO with ChromeGCN for 103 chromatin features on GM12878 cell line.
