# Supplementary figures and images for: Characterizing collaborative transcription regulation with a graph-based deep learning approach
Source: PLoS Comput Biol. 2022 Jun 6;18(6):e1010162. doi: 10.1371/journal.pcbi.1010162 (PMC9203014; doi:10.1371/journal.pcbi.1010162)

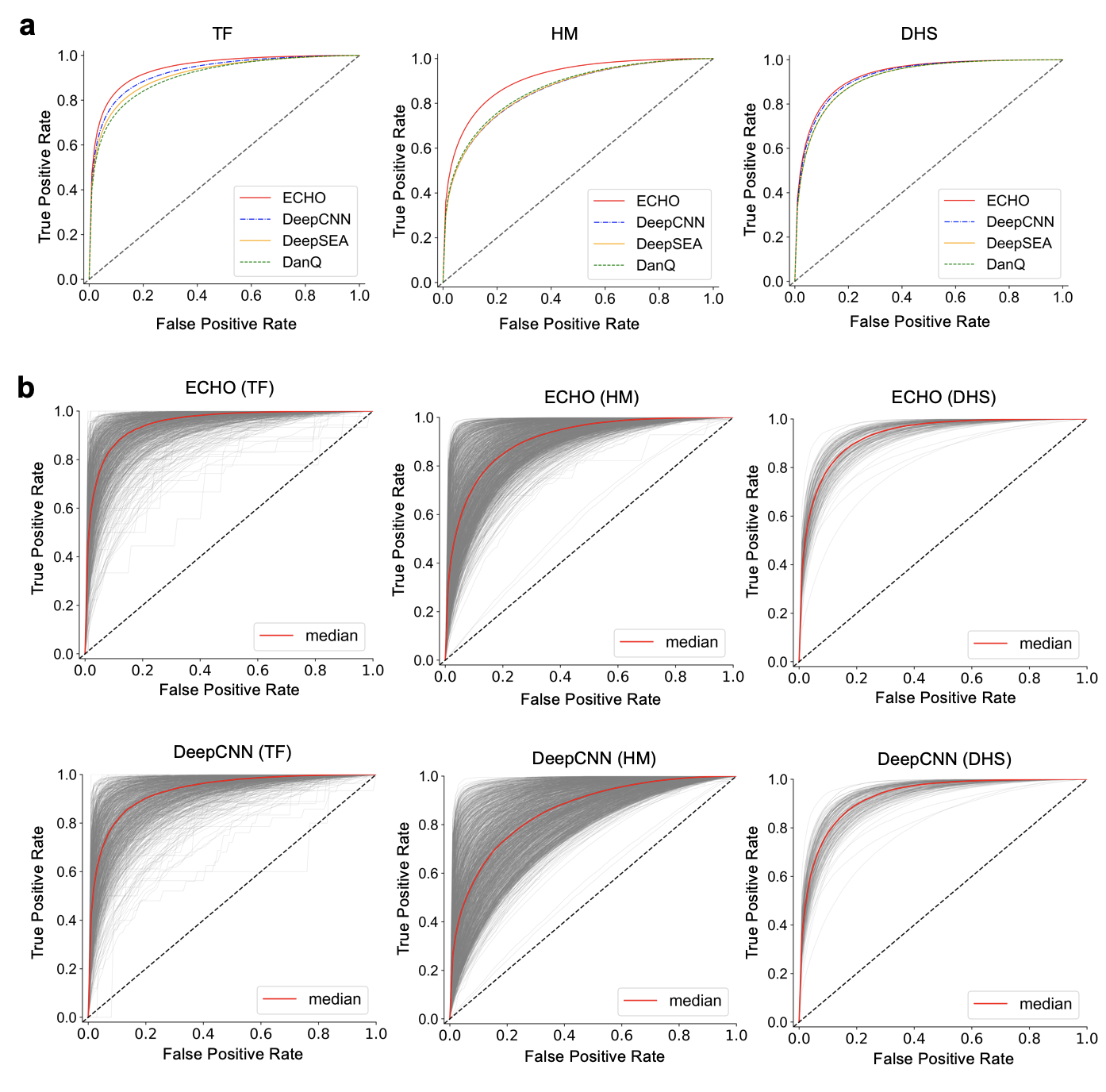

Supplement: S1 Fig — (a) The mean ROC curves from ECHO and three baseline models for three types of chromatin features, including TF, histone mark and DHS. ECHO achieves higher mean AUROC scores than the baselines, especially on TF and histone mark. (b) The ROC curves for each chromatin feature from ECHO and DeepCNN models. The red lines denote the median ROC curves. (TIF) [file pcbi.1010162.s004.tif]

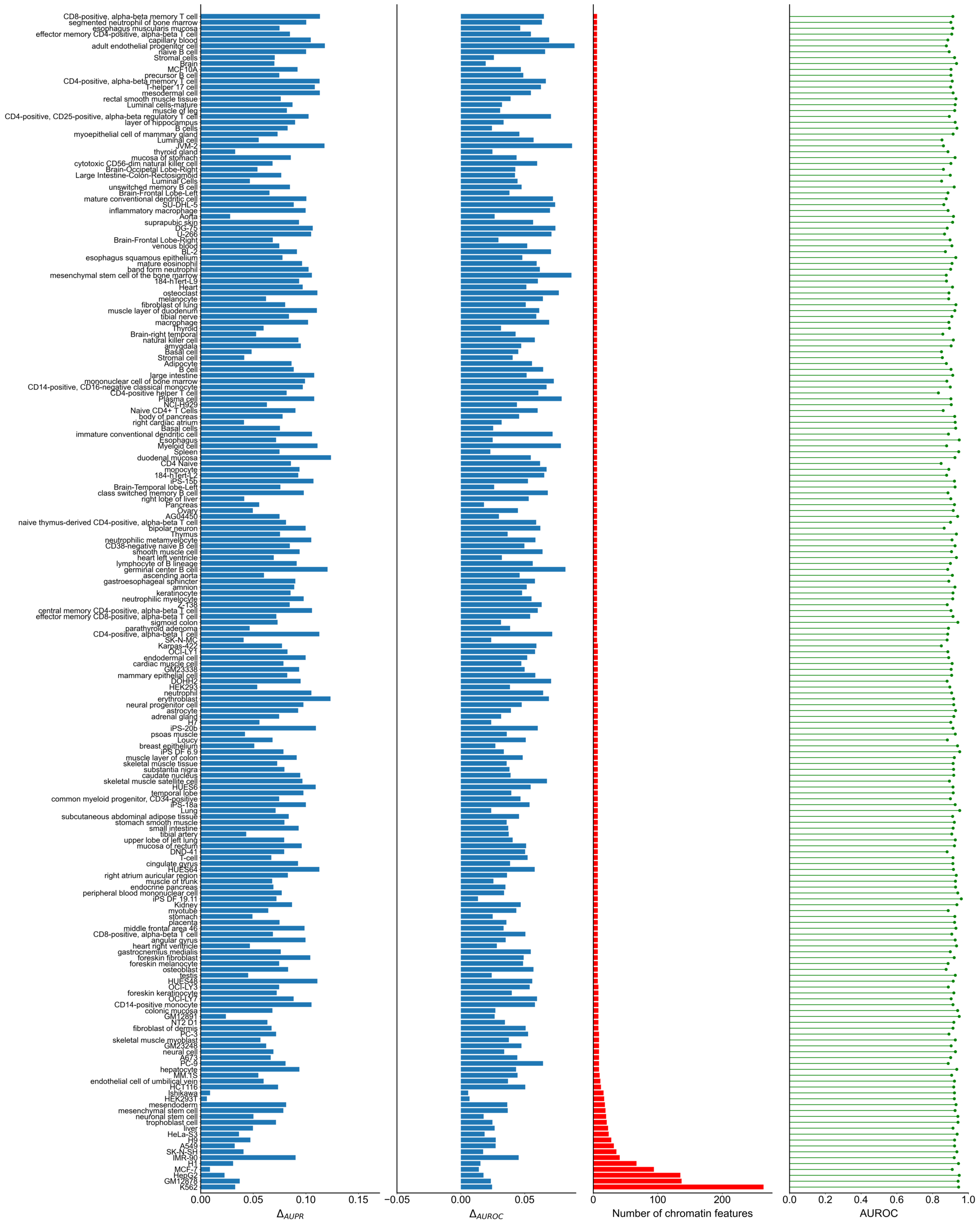

Supplement: S2 Fig — The results of 200 cell lines with most collected chromatin feature profiles are provided, the rest cell lines are shown in S3 Fig. The first column shows the improvement on mean AUPR score for each cell line, the second column shows the improvement on mean AUC score, the third column displays the number of collected chromatin features, and the fourth column shows the mean AUROC scores. (TIF) [file pcbi.1010162.s005.tif]

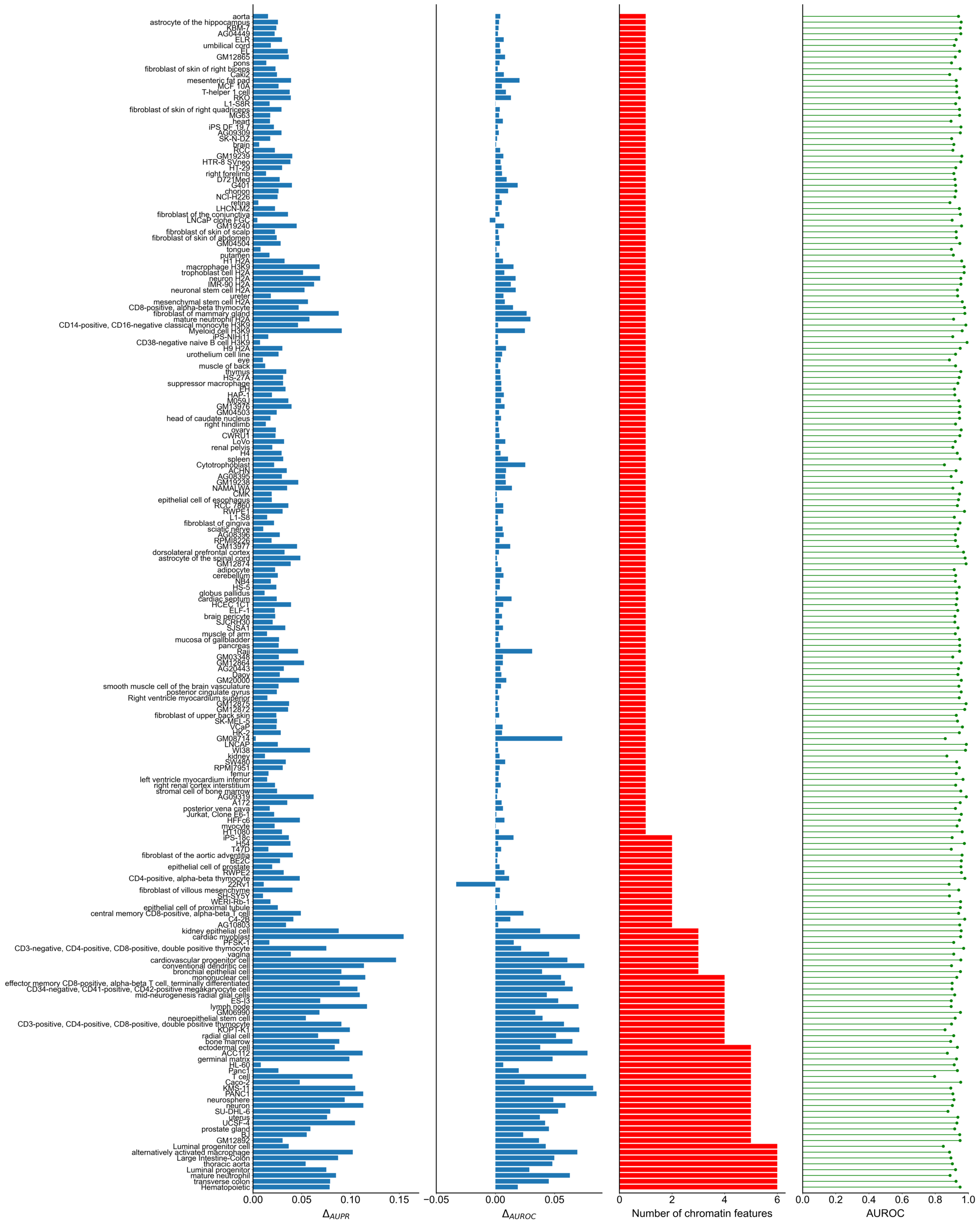

Supplement: S3 Fig — The results of the rest 202 cell lines with least collected chromatin features are shown. (TIF) [file pcbi.1010162.s006.tif]

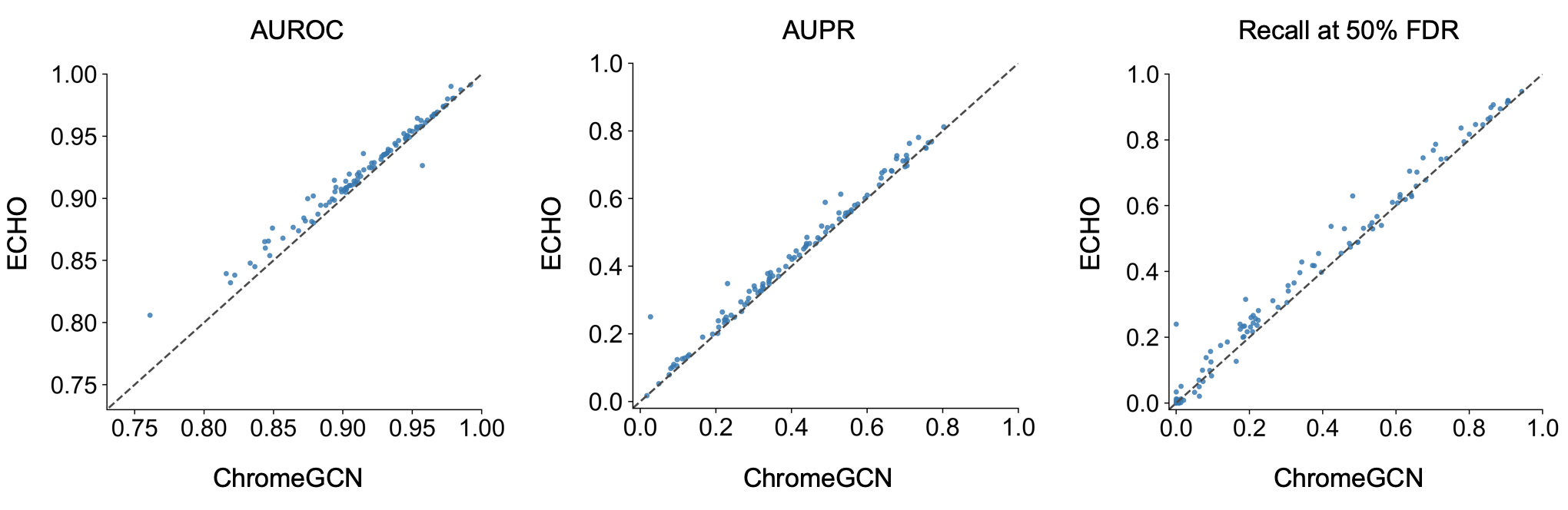

Supplement: S4 Fig — (TIF) [file pcbi.1010162.s007.tif]

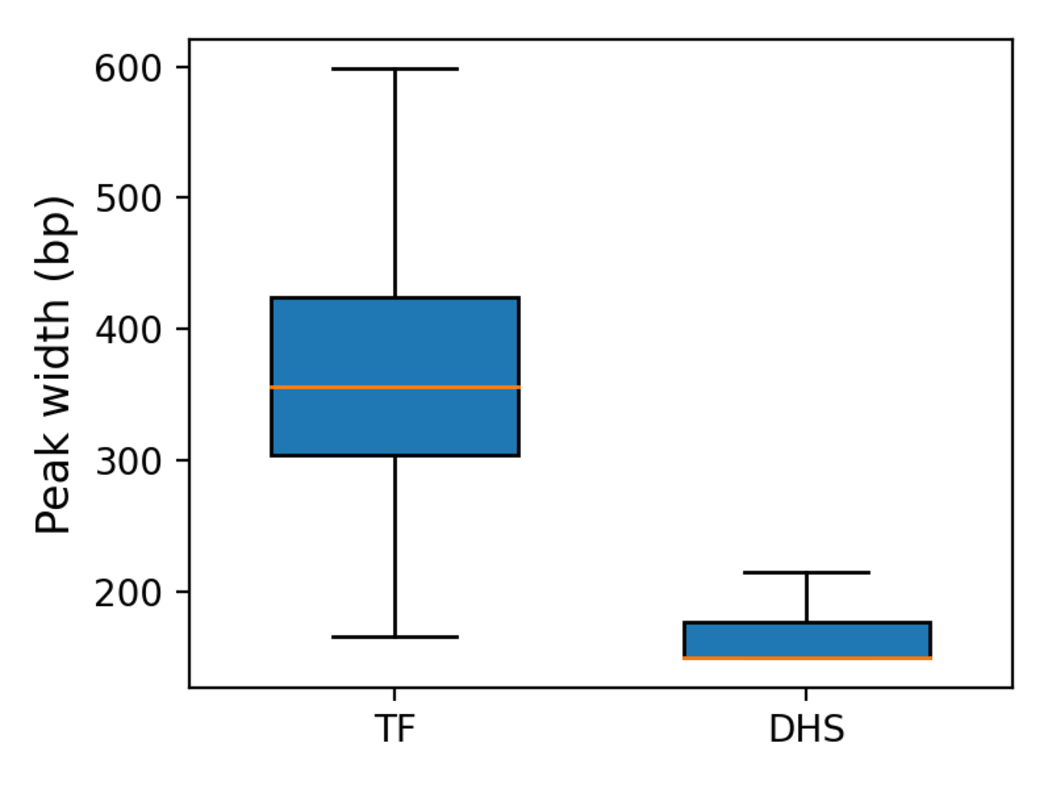

Supplement: S5 Fig — The mean DHS peak width is 162bp and the mean TF peak width is 383bp. (TIF) [file pcbi.1010162.s008.tif]

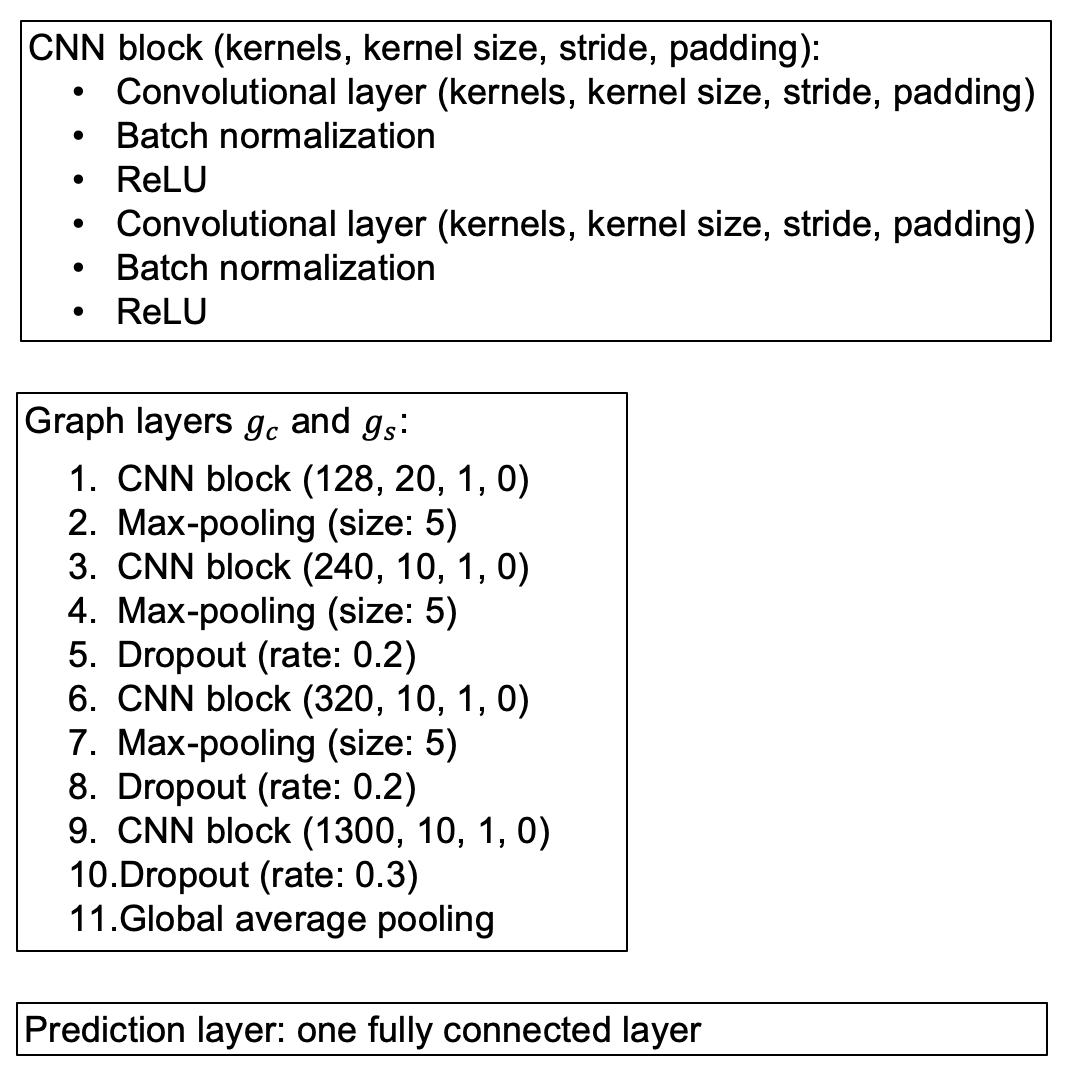

Supplement: S6 Fig — The architectures of graph layers are varied considering the number of chromatin features, the input sequence size, and whether sequential neighbors are sampled. The model architecture reported here is for predicting 2,583 chromatin features with 50 spatial neighbors and 10 sequential neighbors per input sequence. (TIF) [file pcbi.1010162.s009.tif]

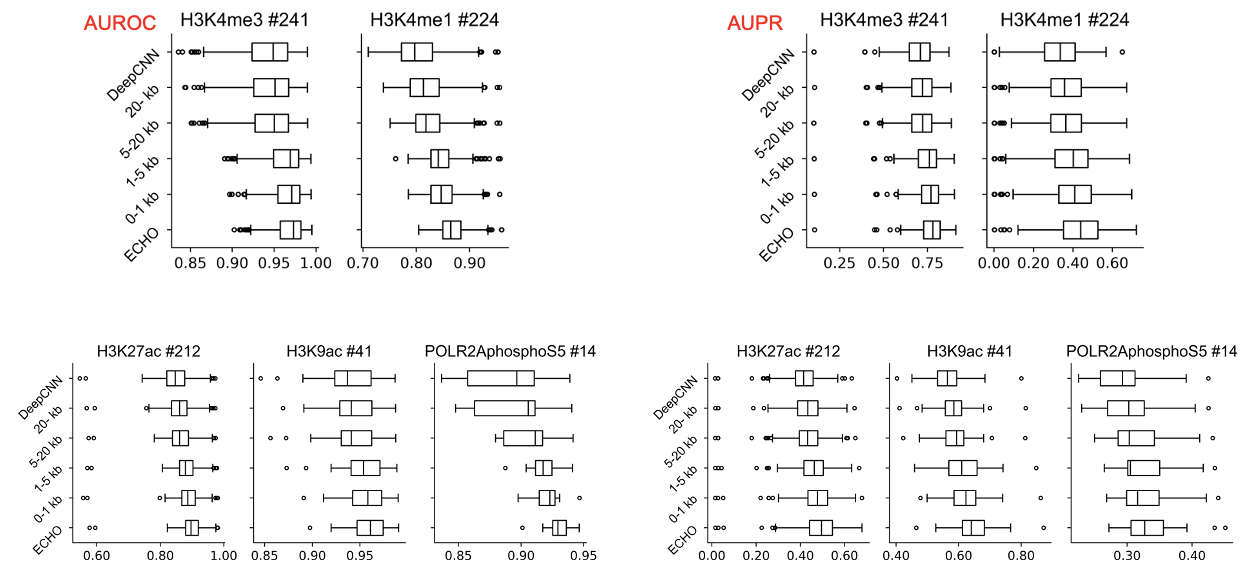

Supplement: S7 Fig — (TIF) [file pcbi.1010162.s010.tif]

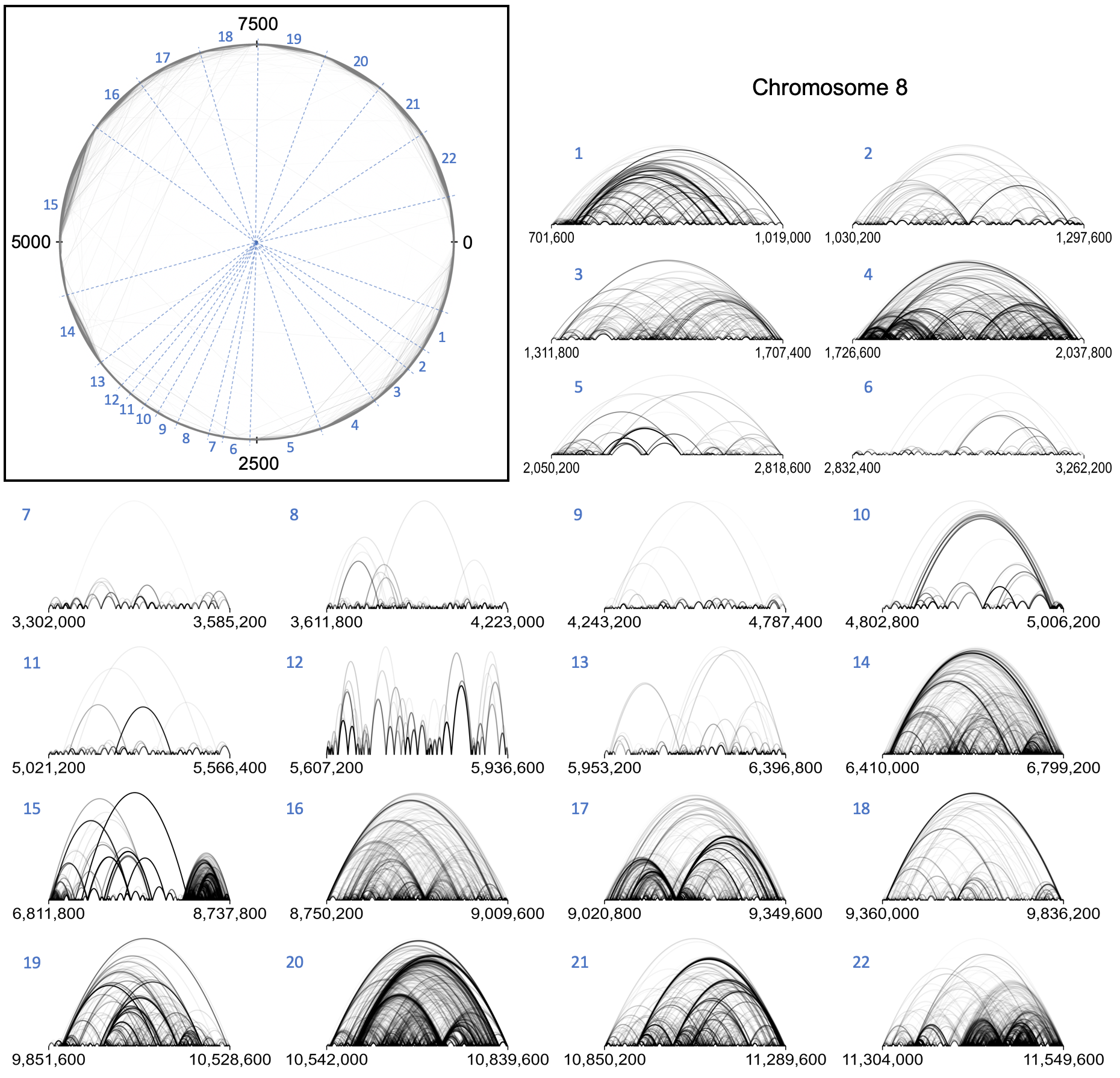

Supplement: S8 Fig — The Mirco-C contacts within the first 10k sequences in Chromosome 8 are visualized in a circle. 0.988 of the total attribution scores for all chromatin features are total attribution scores of contacts within TADs, and 0.982 of the contacts are in TADs. The blue dashed lines show the hESC TAD boundaries. The black numbers on the circle index the 10k sequences, and the blue small numbers index the 22 TADs. The attribution scores of contacts for all chromatin features within each TAD are plotted. The color transparency of the lines represents the values of attribution score. (TIF) [file pcbi.1010162.s011.tif]

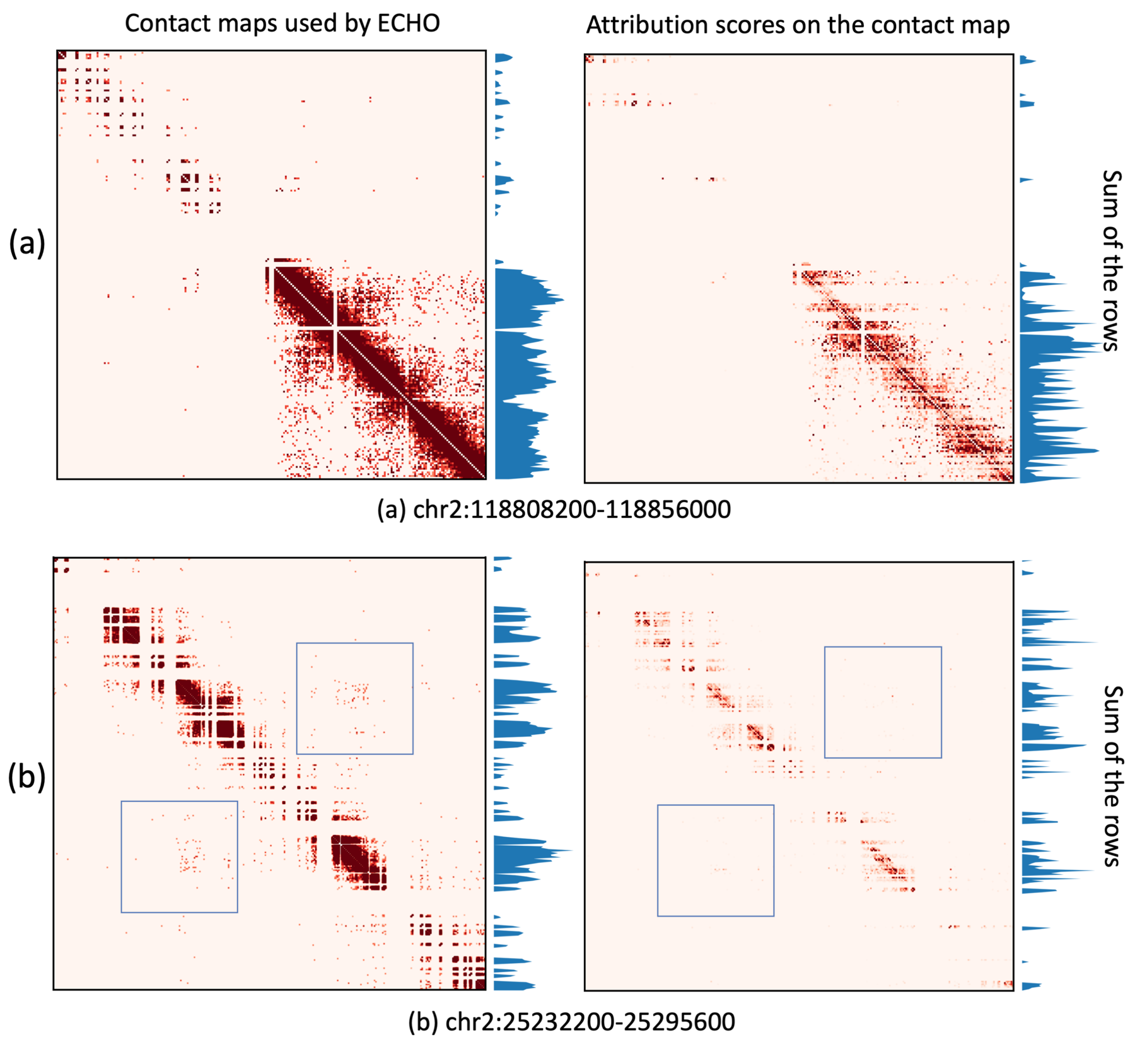

Supplement: S9 Fig — Two example regions in H1 cell line are provided. The original contact matrices are shown in the left column and the yielded attribution matrices from ECHO are shown in the right column. (a) A region where the attribution scores resemble the original chromatin contact matrices. (b) In a region where the attribution scores are different with the contact matrices, the two blue squares in the figure show the chromatin contact patterns which are not reflected by the attribution scores. The chromatin contact matrices are symmetric whereas the attribution score matrices are asymmetric with (i, j)−th entry denoting the importance of sampling neighbor sequence j to the chromatin feature prediction on the central sequence i. Sum of the rows are provided on the right of each matrix. Notice that the chromatin contact matrices are populated for every 200bp sequence, but the attribution scores can be zero by default for the entire row if the 200bp sequence is not used by ECHO (ECHO only used the genomic regions with at least one TF binding events in all used cell lines, following the same strategy used by DeepSEA) or no chromatin features in H1 cell line appear in the 200bp sequence. (TIF) [file pcbi.1010162.s012.tif]

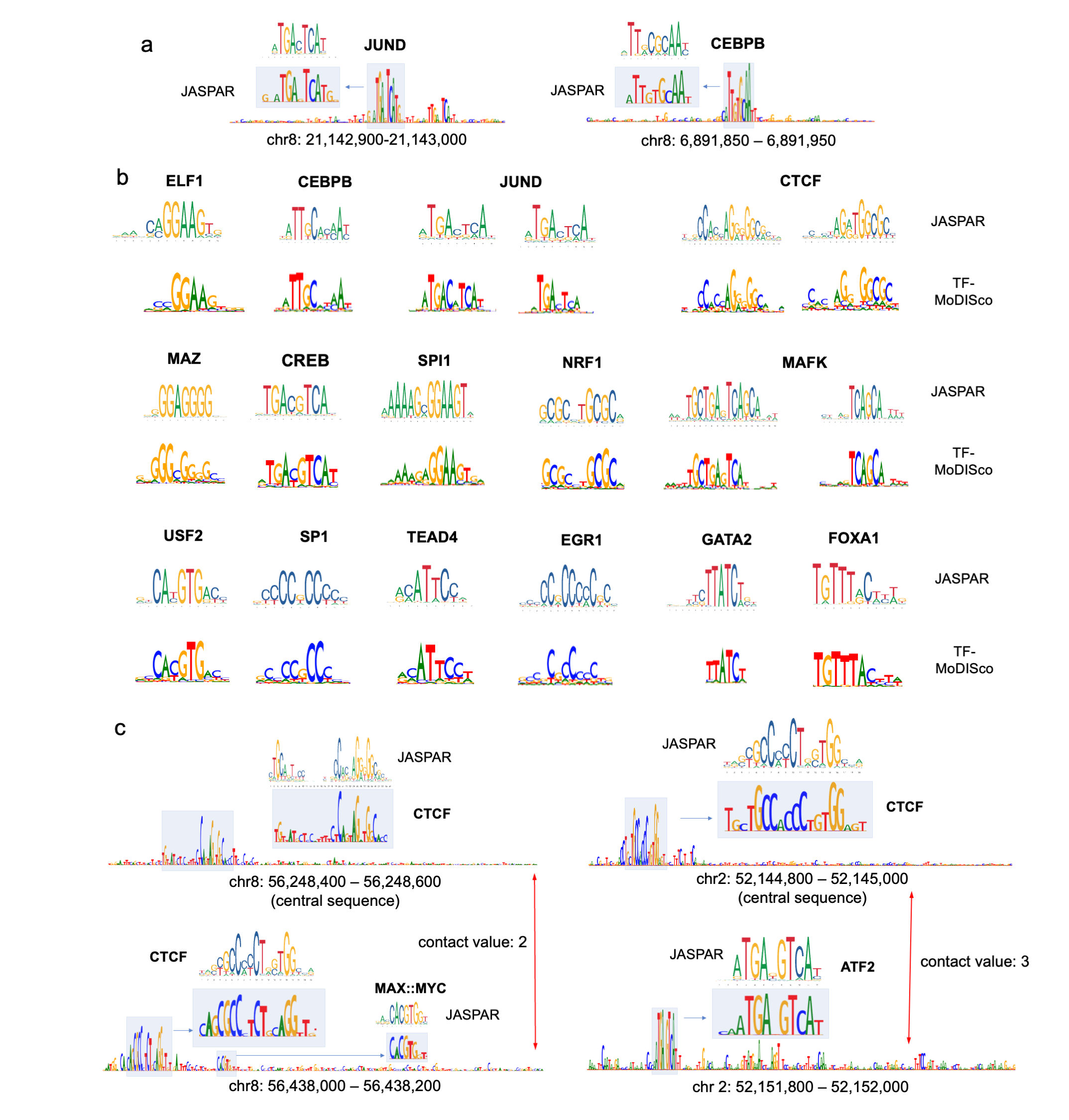

Supplement: S10 Fig — (a) Attribution scores of DNA sequences for two specific TFs, JUND and CEBPB. The height of each letter (A,T,C,G) shows the attribution score for the exact base pair. The high score regions are compared with known motifs from the JASPAR database [24]. (b)Sequence patterns generated by TF-MoDISco [25]. For each TF, the sequence patterns are generated from the attribution scores of 100 binding sites which are also successfully predicted by ECHO. These patterns match the known binding motifs from JASPAR. (c)Attribution scores of the central sequences and the neighbor sequences toward TF binding on central sequence. The Micro-C contact values between central sequences (top) and neighbor sequences (bottom) are given. The high attribution score regions in the central sequence reflect TF binding motifs, whereas the high attribution score regions in the neighbor sequence contribute to the TF binding prediction on the central sequence. The correlated high attribution score regions reveal the potential collaborative binding mechanisms of TFs. For example, we observe a CTCF pattern in the central sequence, and a CTCF pattern and a MAX::MYC pattern in the neighbor sequence. Our observation agrees with the previous study that CTCF and MAX which frequently exist at the chromatin loop anchors may form a complex and participate in CTCF loops [39]. (TIF) [file pcbi.1010162.s013.tif]

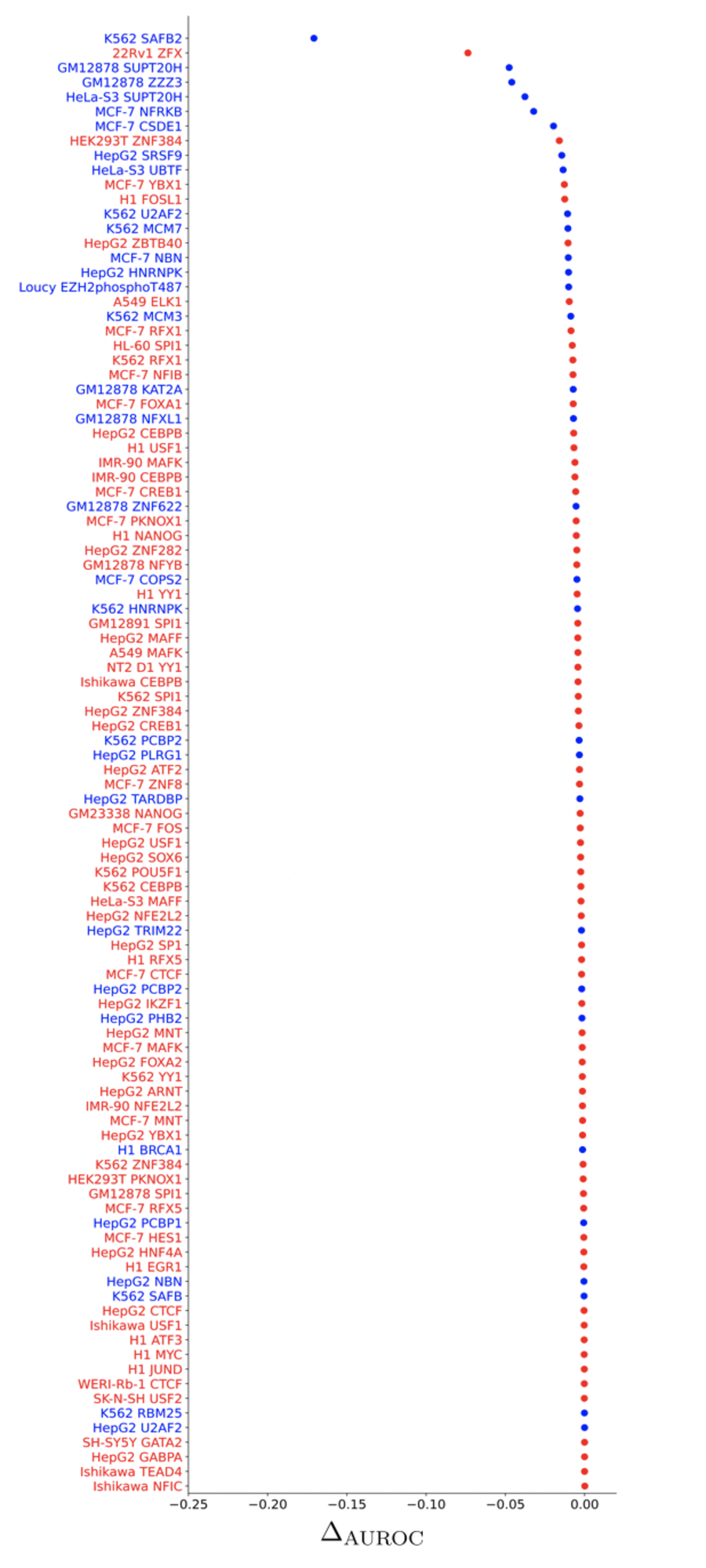

Supplement: S11 Fig — TFs without known motifs from JASPAR are marked in blue, others are marked in red. (TIF) [file pcbi.1010162.s014.tif]

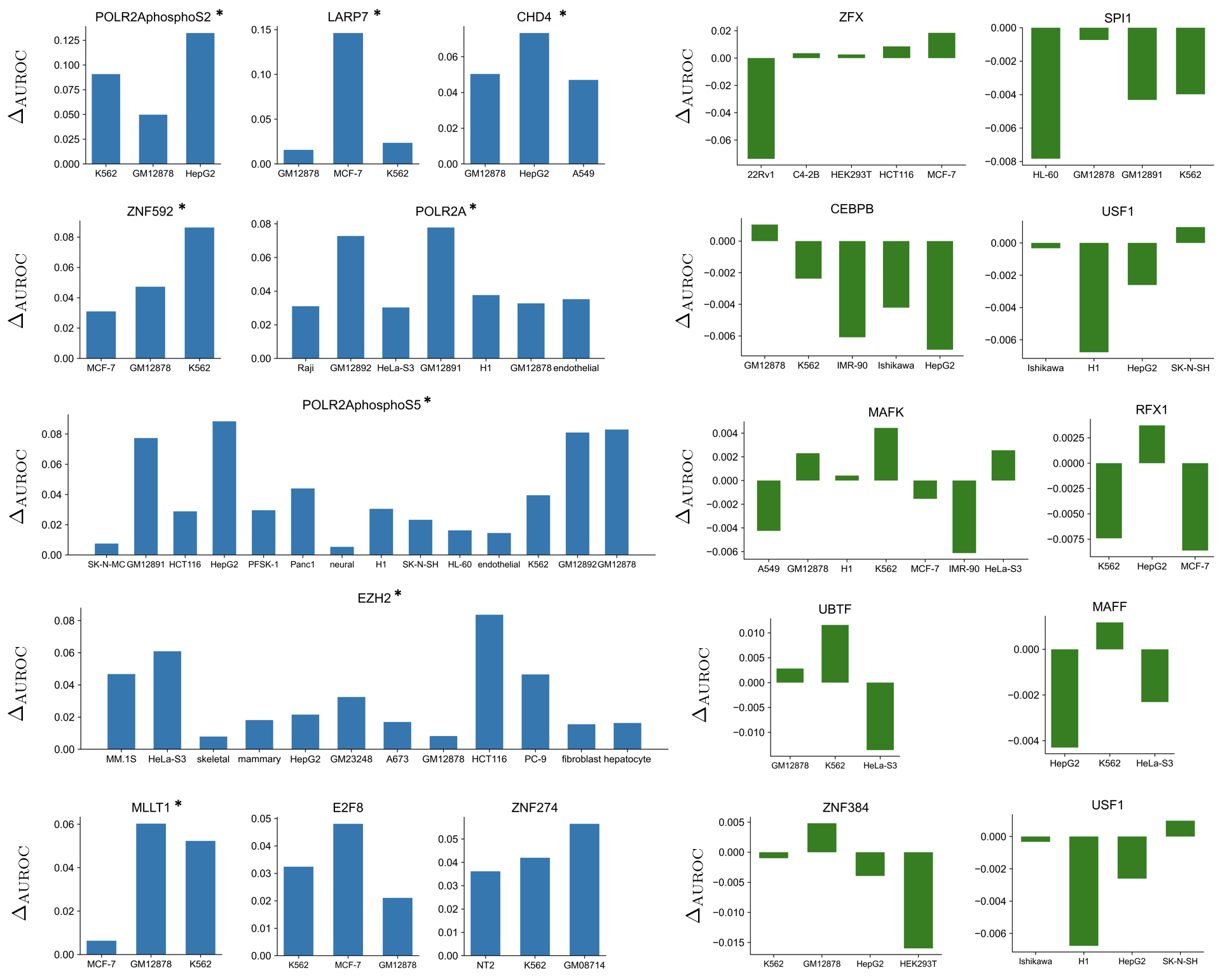

Supplement: S12 Fig — We identify ten TFs for which ECHO and DeepCNN predict quite differently among more than three cell lines. The Y-axes show the differences of AUROC scores (AUROC from ECHO minus AUROC from DeepCNN). TFs without known motifs are marked with ‘*’. (Left panels) TFs whose AUROC scores are significantly higher in ECHO than DeepCNN. (Right panels) TFs whose AUROC scores are slightly higher or lower in ECHO than DeepCNN. (TIF) [file pcbi.1010162.s015.tif]
