# Supplementary material for: Characterizing collaborative transcription regulation with a graph-based deep learning approach
Source: PLoS Comput Biol. 2022 Jun 6;18(6):e1010162. doi: 10.1371/journal.pcbi.1010162 (PMC9203014; doi:10.1371/journal.pcbi.1010162)
Supplement: Attachment — Submitted filename: Response.pdf [file pcbi.1010162.s016.pdf]

Response to Reviewers

Thank you for reviewing our manuscript titled "Characterizing collaborative transcription regulation with a graph-based deep learning approach". Below, we address each of the points raised by the three reviewers and describe the changes we have made to the manuscript. In what follows, the reviewer's comments are given in black, interleaved with our responses in blue, and major changes of the main text in red.

Reviewer #1

The authors developed this new computational method called ECHO for the prediction of chromatin features by using DNA sequence and Micro-C contact maps as input. As compared to the existing method that explicitly considers the chromatin interactions, ECHO introduced computational techniques in GraphSAGE, which allows the model to be trained on graphs at a large scale, i.e. Micro-C data at high resolutions. The authors demonstrated that ECHO has improved performance for predicting chromatin features, and by applying gradient-based attribution methods, important contacts and sequence features can be identified. However, I have some concerns in terms of the performance evaluation and analysis results. See my detailed comments below:
Major:

1. One important thing that the authors should clarify in the motivation of this work is why predicting chromatin features at high-resolutions (200bp) is important. Since one of the major contributions of this work is improved scalability of the method on a large-scale chromatin interaction network, the authors should justify that this problem indeed exists. Perhaps the author could report the average width of the peaks called from these chromatin features? What's the typical size of the narrow peaks and broad peaks, can they already be captured well with the original 1kb resolution used in chromeGCN?

> Thank you for your suggestions. We agree with you that it is quite important to motivate predicting chromatin features at high-resolution (200bp). Following your advice, we investigated the average width of the peaks called from these chromatin features, and observed that the widths of narrow peaks downloaded from ENCODE were at hundreds-of-base-pair scale (Fig R1, the average peak widths of DHSs and TFs were 162bp and 383bp, respectively). These facts suggested that it was desirable to predict chromatin features at high-resolution (200bp). Indeed, previous chromatin feature prediction models such as DeepSEA and DanQ also predicted chromatin features at 200bp resolution (namely a 200bp central region and two 400bp flanking regions both upstream and downstream). In addition, due to special splicing enzymes used, Micro-C cuts chromatin into single nucleosomes (rather than into a few nucleosomes in Hi-C) and measures chromatin organization at 200bp resolution, which makes it possible to leverage chromatin organization in predicting chromatin features at 200bp. Following your advice, we revised our manuscript as follows.
>
> "(Line 92) Additionally, ECHO utilized 200bp resolution Micro-C contact maps since many of the DHS peaks were narrower than 200bp, and the widths of TF binding sites were typically much smaller than 200bp (Fig S5). To predict chromatin features at this high resolution, Micro-C which detected chromatin contacts between much shorter fragments than Hi-C and better illustrated fine-scale chromatin interactions, was more desirable."

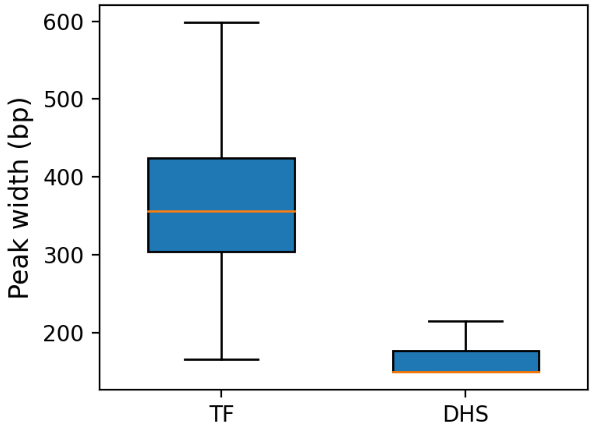

Fig R1: A box plot of average peak widths of TFs and DHSs.
The mean DHS peak width is 162bp and the mean TF peak width is 383bp.

2. Some related works are discussed but not included in the performance comparisons. The authors briefly discussed SATORI, Basenji, and Enformer in the discussion part, but did not include any of them in the performance comparisons. The authors mainly compared ECHO against DNA sequence models with small flaking regions being considered. It is important to show that ECHO reflects the collaborative binding mechanisms among TFs that these new DNA-sequence-based models with larger receptive field sizes could not capture.

Thank you for your comments. Basenji and Enformer do not intend to capture TF collaborative binding activities. Although collaborative binding mechanisms can be potentially captured by the attention strategy used by Enformer and the dilated convolution strategy used by Basenji, the collaborative binding activities that can be potentially recovered by these models are limited within their receptive fields. In addition, the work of Enformer mainly uses attention matrices and attribution methods to explain CAGE-seq prediction, and identifies active enhancers rather than collaborative binding mechanisms. In the work of SATORI, attention mechanism is used to learn the interactions between learned TF motifs on DNA sequences, but it only captures collaborative binding in a short range (within 1kb distance). To sum up, the collaborative binding activities to be captured are not the focus of these models, and even if they can be captured, they are limited to a small region.

By contrast, ECHO leverages Micro-C contact maps and identifies collaborative bindings guided by the contact maps, without a range limit. For example, we first found that potential CTCF interactions with contact distances greater than 100kb contribute to CTCF binding prediction (Fig R3). An example of these CTCF interactions with attribution scores on both anchors, which reflects collaborative binding, has been provided in Fig R2 (Fig S10c in the revised manuscript), and the contact distance of the interaction is 190kb which cannot be captured by Enformer. In this example, the attribution scores on both the central sequence and the neighbor sequence were calculated based on TF binding on the central sequence. We observed a CTCF pattern in the central sequence, and a CTCF pattern and a MAX::MYC pattern in the neighbor sequence that contributed to CTCF binding prediction. CTCF and MAX which frequently exist at loop anchors may form a complex and participate in CTCF loops [1], so the correlated high attribution score regions on the central and neighbor sequences may reflect CTCF collaborative binding. We have added the comparison in the Results section which discusses the collaborative binding mechanisms as follows.

“(Line 249) Previous chromatin feature prediction models like Enformer and Basenji did not focus on capturing TF collaborative binding. Although collaborative binding mechanisms could be potentially captured by the attention strategy used by Enformer and the dilated convolution

strategy used by Basenji, the collaborative binding activities that could be potentially recovered by these models were limited within their receptive fields (e.g., up to 100kb in Enformer). Another prediction model SATORI could capture collaborative binding but it was limited to a small region (within 1kb distance). By contrast, ECHO leveraged Micro-C contact maps and could identify collaborative bindings guided by chromatin contact maps, without a range limit. The collaborative binding mechanisms were reflected by the attribution scores calculated on both the central sequence and its neighbor sequences related to chromatin features on the central sequence. We first found that the highly attributed regions on the central sequences matched known binding motifs from JASPAR (Fig S10a). Furthermore, some high attribution score regions in the neighbor sequences which contributed to chromatin feature prediction on the central sequence, also matched known motifs from JASPAR. Therefore, the correlated high attribution score regions on both central and neighbor sequences might reflect TF collaborative binding (Fig S10c, and the first example showing collaborative binding patterns with a distance of 190kb)."

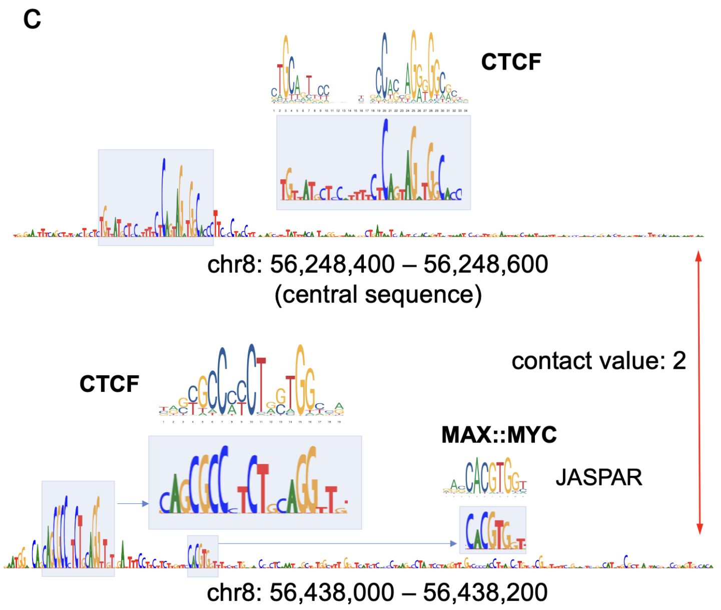

Fig R2: Attribution scores of the central sequences and the neighbor sequences toward TF binding on central sequence. The Micro-C contact values between central sequences (top) and neighbor sequences (bottom) are given. The high attribution score regions in the central sequence reflect TF binding motifs, whereas the high attribution score regions in the neighbor sequence contribute to the TF binding prediction on the central sequence. The correlated high attribution score regions reveal the potential collaborative binding mechanisms of TFs. For example, we observe a CTCF pattern in the central sequence, and a CTCF pattern and a MAX::MYC pattern in the neighbor sequence. Our observation agrees with the previous study that CTCF and MAX which frequently exist at the chromatin loop anchors may form a complex and participate in CTCF loops [1].

3. Some of the analysis results are not rigorous enough. For instance, the authors observed that for bins with CTCF binding events, the proportion of their associated contacts where the other anchor was also bound by CTCF increases by the genomic distance. The authors then reached the conclusion that "CTCF interactions had more effects on CTCF binding as contact distance increased". Such conclusion is strange as CTCF binding happens before the formation of CTCF loops. The main drawback of such an analysis process is that the attribute scores based on gradient only suggest "correlations" or "dependencies" not "causality". It is known that for loops or significant contacts, the ratio of CTCF loops increases with the distance. Thus, naturally, loops of longer distance would contribute more to CTCF binding predictions. The latter relationship between the distance of loops and CTCF binding is caused by the confounding factor of

the relationship between the ratio of CTCF loops and distance.

Thank you for your comments. We agree with you that our conclusion "CTCF interactions had more effects on CTCF binding as contact distance increased" is strange, but we feel that we did not make it clear in our original manuscript and caused some confusion.

We understood that the ratio of CTCF loops increased with the distance as you mentioned, so we had added a baseline which counted the percentage of interactions whose both anchors were bound by CTCF without being filtered by the attribution scores. The results were plotted as the group 'all' in the Fig R3c (which is Fig 4c in the manuscript), and the percentage indeed increased with the chromatin contact distance. However, its increasing rate was significantly lower than the increasing rates of the percentages of CTCF interactions stratified by attribution scores, which could be observed from the other four groups filtered by attribution scores in the same figure. Therefore, we revised the conclusion sentence as "as the contact distance increased, long-range CTCF interactions were more essential to CTCF binding prediction". We hope our revised text below is not confusing any more.

"(Line 224) Additionally, we added a baseline which identified all Micro-C contacts whose central sequence bound by CTCF without filtering the attribution scores, and obtained the percentages of interactions whose both anchors were bound by CTCF, which were shown as 'all' in Fig 4c. We observed that the percentage of potential CTCF interactions increased with the chromatin contact distance. However, the increasing rate was significantly lower than the increasing rates of the percentages of CTCF interactions stratified by attribution scores, which could be observed from the other four groups filtered by attribution scores in the same figure. Within short-range interactions, the percentages of CTCF interactions were significantly lower than those within long-range interactions. However, as the contact distance increased, long-range CTCF interactions were more essential to CTCF binding prediction."

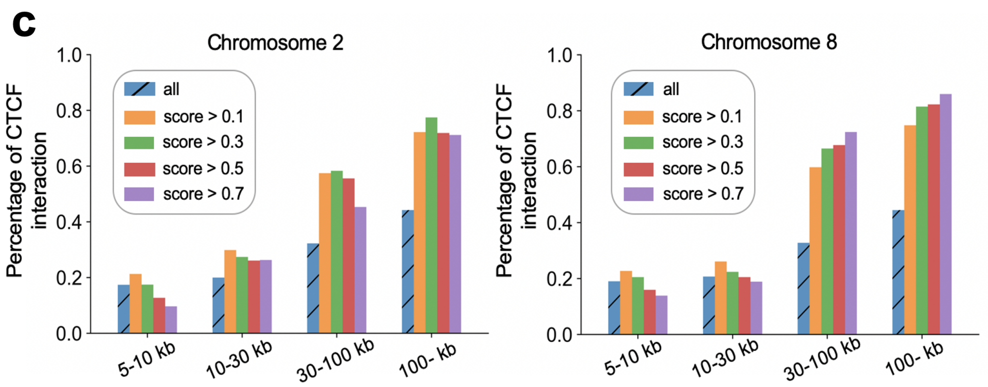

Fig R3: Original Fig 4c copied from the manuscript here for convenience. Micro-C contacts are separated into four groups according to the contact distances: 5−10 kb, 10−30 kb, 30−100 kb, and 100− kb. In each group, the contacts with CTCF related attribution scores greater than 0.1, 0.3, 0.5, and 0.7 are selected, and group 'all' is a baseline where contacts are not filtered by attribution scores. (c) Bar-plots to show the percentage of CTCF interactions, which is calculated as the number of contacts whose two sides were bound by CTCF over the total number of contacts whose central sequence was bound by CTCF.

Minor:

1. I found it hard to understand the following sentence "As a comparison, we computed attribution scores by using DeepCNN who could only attribute each sequence's binding TFs to itself, so the ZBTB3 binding motif was not detected as expected." I suppose the authors mean that "DeepCNN did not utilize Micro-C contacts and thus can not identify important sequence features in its spatial neighbors?"

Yes, it is exactly what we meant — DeepCNN can only attribute the predicted binding TFs to its central sequence because it does not utilize Micro-C contacts. Here we wanted to show that the attribution scores of DeepCNN could miss some binding motifs of unpredicted TFs, but ECHO might detect these binding motifs in the neighbor sequences by identifying important regions in the neighbor sequences which contributed to TF binding prediction on the central sequence. In our experiment, we first observed a ZBTB3 motif in the neighbor sequence which contributed to the TF binding prediction on the central sequence by using ECHO, but ZBTB was not predicted (ZBTB3 could not be directly attributed to the sequence). To test whether DeepCNN could detect this motif, we directly attributed the sequence which contained the ZBTB3 motif based on its predicted binding TFs by using DeepCNN, and as expected this motif was not reflected by the attribution scores from DeepCNN. We have revised our manuscript as follows.

"(Line 277) First, we identified the binding motif of ZBTB3 which frequently appears in the proximity to CTCF [27], from the neighbor sequence's attribution scores, but we found that ZBTB3 was not selected in our TF profiles (i.e., ZBTB3 was not predicted). Since sequence-based models such as DeepCNN could only attribute the predicted binding TFs to its central sequence, this motif might not be detected from DeepCNN's attribution scores. To test if DeepCNN could detect this motif, we directly attributed the sequence which contained the ZBTB3 motif based on its predicted binding TFs by using DeepCNN, and the ZBTB3 binding motif was not detected from the attribution scores as expected. Therefore, DeepCNN's attribution scores could miss some binding motifs of unpredicted TFs, but ECHO might detect these binding motifs in the neighbor sequences by identifying important regions which contributed to TF binding prediction on the central sequence."

Reviewer #2

In this paper, Zhang et al. aim to better characterise the collaborative activity of the epigenome mediated through 3D interactions. To achieve this, they developed the ECHO model, which leverages both DNA sequence and Micro-C data to predict the absence or presence of epigenomic features for the region of interest across different cell lines.

To evaluate ECHO's performance, the authors compared it to sequence-only models (Deep-CNN, DeepSea and DanQ) as well as one alternative model incorporating spatial information (ChromeGCN). The authors showed how ECHO out-performs all considered models, with regards to AUROC and AUPR metrics, to varying degree depending on the predicted feature of interest. More specifically, they observed a dramatic improvement for histone modifications, a moderate improvement for transcription factors and a mild improvement for Dnase hyper-sensitive sites. Nevertheless the improvement was not obvious for some underrepresented cell types. They also showed that the influence of pre-trained sequence-feature layers was only marginal, indicating the decisive contribution of the spatial components of ECHO. The authors also evaluated the influence of factors specific to chromosome-capture sequencing data and found important and varying effects on model performance depending on the linear distance-range of the interactions considered as well as on the bin size of the interaction data.

The authors also demonstrate the unique insights captured by ECHO because of its consideration for spatial interactions and functional collaborations. They manage to derive such interpretations of the model by using gradient-based attribution methods that trace the contribution of the different components of the model for individual predictions. These attribution methods allowed the authors to examine how sequence information contributed to model prediction while integrating the spatial configuration harnessed by graph layers. Of particular interest, the authors were able to reliably detect long-range CTCF-interactions as well as recover validated binding co-factors in the chromatin neighbourhood of these CTCF binding-sites. Similar properties were found for SMAD5 and H3K4me3.

Comments:

The paper presents an original and timely contribution to the field, while making relevant benchmarkings with recent instances of deep neural networks applied to epigenomic characterisation. The authors also provide a detailed description of the model and algorithm and provide the necessary supporting information online. The manuscript is also well organized and written clearly enough to be accessible to non-specialists.

Nevertheless, there are several points that I found to not be described in enough details and would justify some additional analyses before I recommend this paper for publication.

Major:

I) From the manuscript, it is unclear how redundant the sequential and spatial neighbours were. Especially given that the closest linear neighbours would tend to also be neighbours with the highest Micro-C interaction signal. Could the authors provide a quantitative evaluation of this likely redundancy? If sequential and spatial neighbours were to be extensively redundant, the benefit of the spatial components of ECHO might need to be re-evaluated. A possibly interesting extension, if we were to witness this redundancy, would be to use the observed/expected transformation of Micro-C data instead of the normalized read-counts currently used to sample spatial neighbours. Here "expected" would reflect the expected linear distance decay of Micro-C signal. The observed/expected transform might therefore more distinctly reflect the spatial configuration complementing the sequential organisation of the chromatin.

> Thank you for your suggestions. Following your suggestion, we investigated the redundancy between sequential and spatial neighbors. It turned out that the redundancy level was quite low. On average, seven out of the fifty spatial neighbors were also sequential neighbors. Nevertheless, we added a new experiment in which spatial neighbors were sampled from observed-over-expected (O/E) normalized contact maps. However, we observed that ECHO's performance did not improve when we sampled from the O/E normalized contact maps. The AUROC score was 0.919 and AUPR was 0.374, which were both lower than ECHO's original scores (AUROC 0.921 and AUPR 0.378).

II) To further explore the merits of ECHO's spatial components, maybe more targeted and informative models should be considered when reporting model performance using the AUROC and AUPR metrics. An example that comes to mind would be to compare the original ECHO model with an ECHO model without a spatial neighbours stack, but a larger set of sequential neighbors only. For example, maybe 60 sequence neighbours split evenly up and downstream of the central sequence. This alternative model would be of a comparable complexity and highlight the exclusive contribution of the spatial stack.

> Thank you for your suggestions. We trained the model with 100 sequential neighbors split evenly up and downstream of the central sequence, and the spatial neighbors were not used. The mean AUROC score was 0.917 and AUPR score was 0.369, which were slightly lower than original ECHO with spatial neighbors sampled (AUROC 0.921 and AUPR 0.378). We have added this result to the Supplementary Table 2 and mentioned it in our manuscript.
>
> "(Line 113) To show the importance of spatial neighbors from contact maps, we built ECHO with only a large number of sequential neighbors along the DNA sequence but no spatial neighbors, and the model yielded an AUROC score 0.917 and an AUPR score 0.369 which were lower than those from original ECHO model (Table S2)."

III) An aspect that wasn't clear in the manuscript was the extent to which the authors were able to recover the cell-specific Micro-C pattern through the attribution method? The reason this might be interesting is that it would indicate the extent to which the model needs to reproduce the 3D configuration of the chromatin in order to recover the main determinants of the predicted epigenomic features. What is the correspondence between spatial proximity and model relevance to predict epigenomic features? Of course such an analysis would only be relevant for the cell-lines for which Micro-C is available, but this would still be informative in further interpreting and explaining the model.

> Thank you for your inspiring comments. First, we do not expect that ECHO's attribution matrices recover the input chromatin organization, because not all of the chromatin contacts are related to the chromatin features to be predicted by ECHO. Nevertheless, your remark inspired us to investigate further. We chose H1 cell line, and attributed the chromatin contacts based on the chromatin features in H1 cell line (if the central sequence has no chromatin feature in H1, then its chromatin contacts will not be attributed). As expected, the attribution scores did not exactly reflect the comprehensive Micro-C patterns across the genome. The attribution

scores showed more similarity with the chromatin contact maps in some regions than the others. Here, we provided two example regions in Fig R4, in one genomic region (a) the attribution scores resembled the original chromatin contact matrices, and in another genomic region (b), the attribution scores and the contact matrices showed less similarity. We also added sentences to analyze the relationships between the contact maps and the attribution scores.

"(Line 239) Furthermore, to test whether the attribution scores related with chromatin features could reflect cell-type specific Micro-C patterns, we calculated the attribution scores on the chromatin contacts related with the chromatin features in H1 cell line whose Micro-C contact map existed, and compared them with the contact map used by ECHO. Since not all of the chromatin contacts were related to chromatin features to be predicted by ECHO, we did not expect to recover the comprehensive Micro-C patterns from the attribution scores. The attribution scores showed more similarity with the chromatin contact maps in some regions than the others. Here, we gave two example regions in Fig S9. In one genomic region, the attribution scores resembled the original chromatin contact matrices, but in the other region, the attribution scores and the contact matrices showed less similarity."

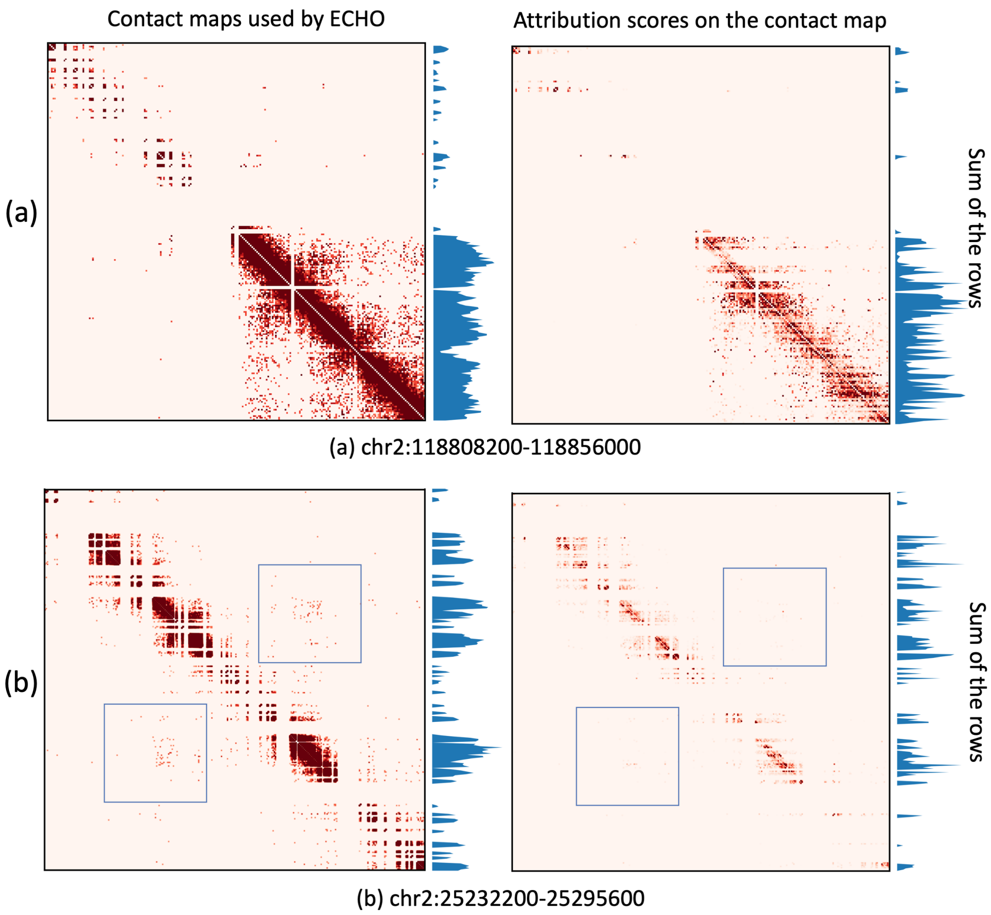

Fig R4: Comparing original contact matrices used by ECHO and the yielded attribution matrices. Two example regions in H1 cell line are provided. The original contact matrices are shown in the left column and the yielded attribution matrices from ECHO are shown in the right column. (a) A region where the attribution scores resemble the original chromatin contact matrices. (b) In a region where the attribution scores are different with the contact matrices, the two blue squares in the figure show the chromatin contact patterns which are not reflected by the attribution scores. The chromatin contact matrices are symmetric whereas the attribution score matrices are asymmetric with $(i, j)-$th entry denoting the importance of sampling neighbor sequence j to the chromatin feature prediction on the central sequence i. Sum of the rows are provided on the right of each matrix. Notice that the chromatin contact matrices are populated for every 200bp sequence, but the attribution scores can be zero by default for the entire row if the 200bp sequence is not used by ECHO (ECHO only used the genomic regions with at least one TF binding events in all used cell lines, following the same strategy used by DeepSEA) or no chromatin features in H1 cell line appear in the 200bp sequence.

IV) It was unclear to me regarding the central regions predicted to be bound by TFs, what was the extent of the recovery of expected known motifs using the attribution methods? This is only described for CTCF, but such evaluation would constitute an important sanity check for the full set of binding factors with known motifs. If the authors could evaluate this, using for example the proportion of such central regions expected to be bound by a factor for which we also recover the corresponding motif through model attribution, it would greatly support the biological relevance of the features produced by ECHO.

Thank you for your suggestions. Since we used TF-MoDISco [2] and manually checked and generated the motifs for each TF from the attribution scores, it is difficult for us to check for the full set of binding factors with known motifs. Here, we provided additional fifteen examples

in Fig S10b in addition to CTCF, these patterns all recovered the binding motifs from JASPAR which were shown in Fig R5. We also uploaded the codes for computing the attribution scores and generating motifs with TF-MoDISco to our GitHub repo, so that other people can easily use the code to calculate attribution scores and generate the motifs for TFs of their interest.

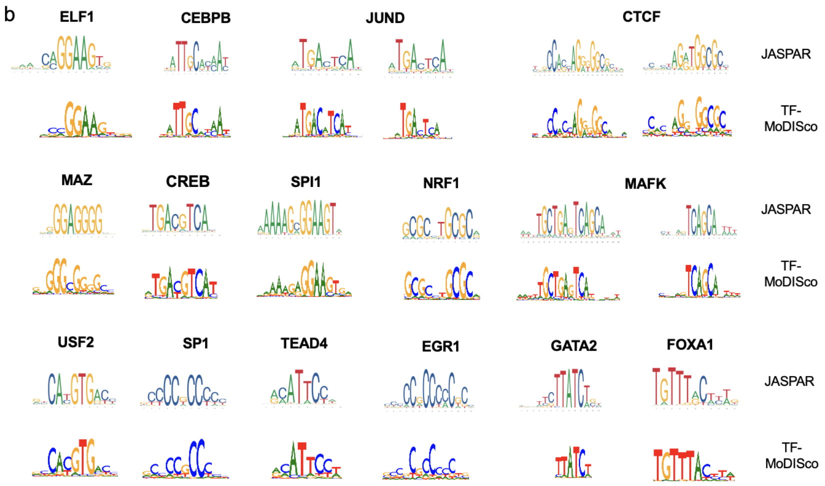

Fig R5: Sequence patterns generated by TF-MoDISco. For each TF, the sequence patterns are generated from the attribution scores of 100 binding sites which are also successfully predicted by ECHO. These patterns match the known binding motifs from JASPAR.

Minor:

I found the comparison with the Enformer model in the Discussion section to be a bit elusive. Since Enformer, like ECHO, leverages a broader context to make its prediction, what would be the exclusive merits of ECHO if instead of predicting gene expression Enformer predicted chromatin features like ECHO?

Thank you for pointing it out: we changed our comparison with Enformer and Basenji, and reorganized the sentences to compare with ChromeGCN in the Discussion section:

"(Line 338) Unlike Enformer and Basenji, ChromeGCN and ECHO leverage 3D chromatin organization which aggregate the regulatory information from the chromatin spatial neighbors without a range limit, so that ChromeGCN and ECHO identify important chromatin contacts contributing to chromatin feature prediction by attributing these chromatin contacts. Furthermore, ECHO identifies collaborative bindings by discovering sequence patterns on both the central sequences and the spatial neighbor sequences, while capturing collaborative bindings is not a focus of Enformer and Basenji. Moreover, ECHO uses Micro-C rather than Hi-C used by ChromeGCN, which better captures fine-scale chromatin contacts and is more desirable to capture interactions between chromatin features. The methodologies used in ChromeGCN and ECHO are also quite different, ChromeGCN converts the entire DNA sequence into the graph structure by leveraging Hi-C contact maps, and uses a gated graph convolution network (GCN) to aggregate the neighborhood information while the gated function determines whether to use the aggregated neighborhood information. Nonetheless, ECHO further transforms the graph structure data from higher-resolution contact maps to grid structure and applies 1D convolutions to learn the neighborhood aggregation patterns."

Reviewer #3

Authors present ECHO, a graph convolutional network that leverages DNA sequence and 3D chromatin architecture derived from chromatin conformation assays to impute epigenomics assays data. This model extends

on widely used sequence based models such as Basset, Basenji, and the recent Enformer by incorporating 3D genome conformation. The ideas behind this method are solid, and authors present convincing improvements on sequence based models that do not explicitly leverage 3D chromatin conformation methods, and also present better performance on a competing model that uses 3D chromatin conformation, ChromeGCN. As the deep HiC/MicroC data needed by the authors for this task is currently only limited to very few cell types, this model is more of a proof of principle but is a useful and promising extension to existing methodologies, and holds great promise for future use. Authors also make efforts to interpret the networks for biological insights and validation of known biological drivers of 3D chromatin conformation, though these efforts are sometimes anecdotal. With some polishing and better presentation of validation/interpretation analyses, this paper is a valuable contribution to the current collection of deep learning models that aim to impute genomic signal.

Major

ECHO's architecture and the concept of using graph convolutions on the graph defined by significant MicroC interactions is an appropriate and refreshing use of 3D chromatin architecture for prediction of chromatin features (using the authors' term) is satisfying.

1. However, I do not understand why sequential neighbors are necessarily included? Presumably short and long range interacting regions that aid the prediction task will be included as per MicroC signal (spatial neighbors). Can the authors explicitly describe the justification of inclusion of 'sequential neighbors' on top of 'spatial neighbors'? One would expect spatial neighbor set to include sequential neighbors. Does the performance of the model suffer dramatically if only 'spatial neighbors' are used?

Thank you for your comments. We believe that sampling sequential neighbors is useful, since the sequential neighbors are the nearest potential TF binding sites to the central sequence, which may affect the central sequences' chromatin features along the DNA sequence. Previous sequence-based models such as Basenji also leverage the sequential neighbors to help predict chromatin features. Moreover, we included a new experiment without sampling the sequential neighbors, and observed that the AUROC and AUPR scores were slightly lower than the original scores of ECHO with both sequential and spatial neighbors (AUROC: 0.918 vs 0.921, AUPR: 0.372 vs 0.378). We have added the new result to the Table S2 and mentioned it in the Results section

"(Line 116) We also showed the importance of sequential neighbors along the DNA sequence by building ECHO with the spatial neighbors only, and the model achieved an AUROC of 0.918 and an AUPR of 0.372 which were sightly lower than those from original ECHO (Table S2)."

and justified why sequential neighbors were sampled in the Methods section as follows.

"(Line 431) Although the sequential neighbors are also likely to be spatial neighbors from Micro-C contact maps, independently sampling the sequential neighbors is able to inform the model that these neighbor sequences are the nearest potential TF binding sites to the central sequence, which may affect the central sequences' chromatin features along the DNA sequence.

2. Contact distance stratified models presented in Figure 2 are interesting. For histone mark prediction, is it possible that histone marks that tend to exhibit broader peaks tend to benefit from short range contacts?

Thank you for your comments. To check whether the hypothesis is correct, we plotted the peak width of each histone mark with different functions and marked the distance range that they benefited from the most in Fig R6. However, we did not observe clear correlation between histone mark peak widths and the contact distances that the histone mark prediction most benefits from. For example, H3K36me3 which benefits most from the 1−20kb contacts, has broader peaks than H3K9ac which benefits most from the 0−1kb contacts.

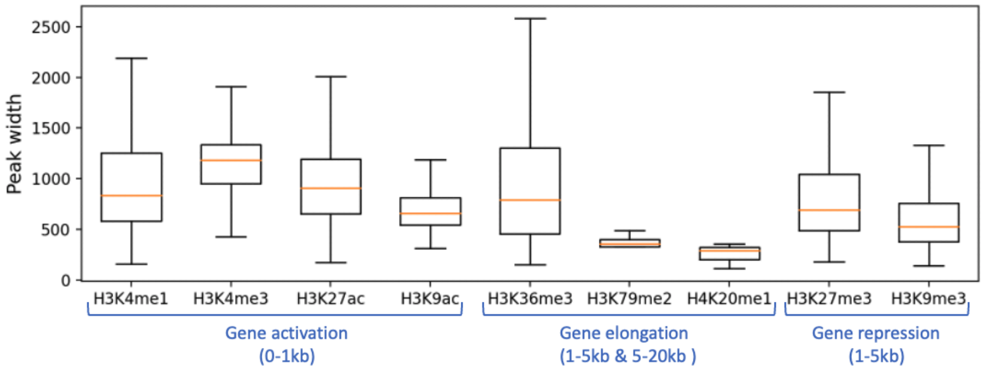

Fig R6: Boxplots of the peak widths of each histone mark with different functions, and the contact distance range that they benefit from the most.

3. The upsampling strategy in "Chromatin contact resolution..." section seems somewhat problematic. More specifically, assuming all 200bp bins will have the same upsampled interaction frequency derived from 1kb Hi-C map is wrong, as I am sure the authors are also aware of. It is clear this strategy is used in this analysis to demonstrate the reduced performance when 200bp resolution interactions are derived from 1kb (HiC) resolution interactions. However, since ECHO uses a sampling strategy for spatial neighbors, would this not mean five 200bp bins that cover a 1kb (HiC resolution) bin with high interaction frequency will be overly represented in the graph structure? In this case, I am unsure if poorer performance results from reduced resolution vs. biased sampling; or these two related effects may be intertwined. I would recommend an alternative analysis; it seems more sensible to me to collapse 200kb MicroC to 1kb resolution without needing any sampling and show that model suffers at this resolution, unless the model is strictly tuned to 200bp resolution. Alternatively, maybe the sampling strategy for lower resolution may be altered.

> Thank you for you comments. Because our model was strictly trained on 200bp resolution, we could not collapse 200bp Micro-C to 1kb resolution. Therefore, we used an alternative sampling strategy for Hi-C — for each 200bp central sequence, we first found the 1kb bin where it located so that we could identify its 1kb spatial neighbors from 1kb resolution Hi-C contact maps. Then, for each of these 1kb spatial neighbors, we connected the centered 200bp bin with the original 200bp central sequence to form a 200-bp resolution Hi-C contact map. The motivation of this alternative sampling strategy was that the centered 200bp bin with the flanking regions could also cover the whole 1kb region (Fig R7). The new results are consistent with the previous results that ECHO with Micro-C achieves higher AUROC and AUPR than ECHO with Hi-C, so high-resolution Micro-C contact maps provide more precise chromatin contact information for chromatin feature prediction. In the manuscript, we have updated the original Fig 3 with the new results (Fig R8) and revised the sentences which described the sampling strategy and reported the new results as follows.

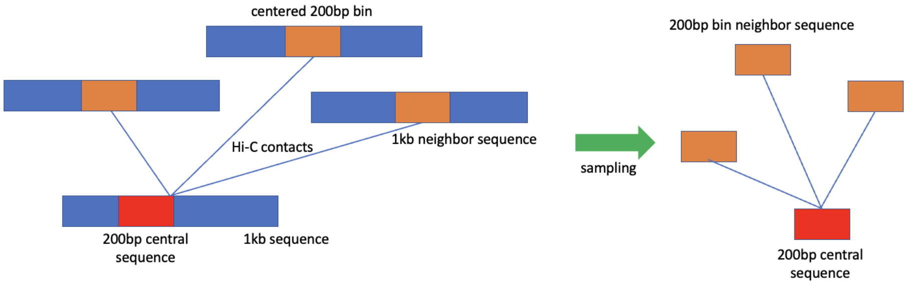

Fig R7: An alternative sampling strategy for a 200bp central sequence (in red) based on its three spatial neighbors from 1kb resolution Hi-C contact map. For each of its spatial neighbors, we connect the central sequence with the 200bp regions in the center (in orange).

> "(Line 186) Since Hi-C could not reach the resolution higher than 1 kb, we sampled 200bp chromatin contacts from the 1 kb resolution Hi-C contact maps as follows. For each 200bp central sequence, we first found the 1kb bin where it located so that we could identify its 1kb spatial neighbors from 1kb Hi-C contact maps. Then, for each of these 1kb spatial neighbors, we connected the centered 200bp bin with the original 200bp central sequence to form a 200-bp resolution Hi-C contact map. The motivation of this sampling strategy was that the centered 200bp bin with the flanking regions could also cover the whole 1kb region. Consistent with the normalization of Micro-C contact maps, both the HFF and H1-hESC Hi-C contact maps were normalized and merged by taking the maximum contact value at each position to generate a weighted adjacency matrix, and contacts with low contact values were filtered out with some threshold. In this experiment, we chose two thresholds and sampled 50 spatial neighbors and 10 sequential neighbors for each sequence similarly, resulting in 61M and 83M Hi-C contacts, respectively. ECHO using Micro-C contact maps predicted chromatin features with a mean AUROC of 0.921, which was significantly higher than ECHO with 83M Hi-C contacts (AUROC 0.914, p-value 7.08E−6), ECHO with 61M Hi-C contacts (AUROC 0.912, p-value 4.67E−10) and DeepCNN (AUROC 0.885, p-value 6.73E−97). Moreover, ECHO with Micro-C contact maps yielded a mean AUPR of 0.378, which outperformed ECHO with 83M Hi-C contacts (AUPR 0.367, p-value 4.92E−2), ECHO with 61M Hi-C contacts (AUPR 0.361, p-value 4.79E−3) and DeepCNN (AUPR 0.318, p-value 7.20E−25)."

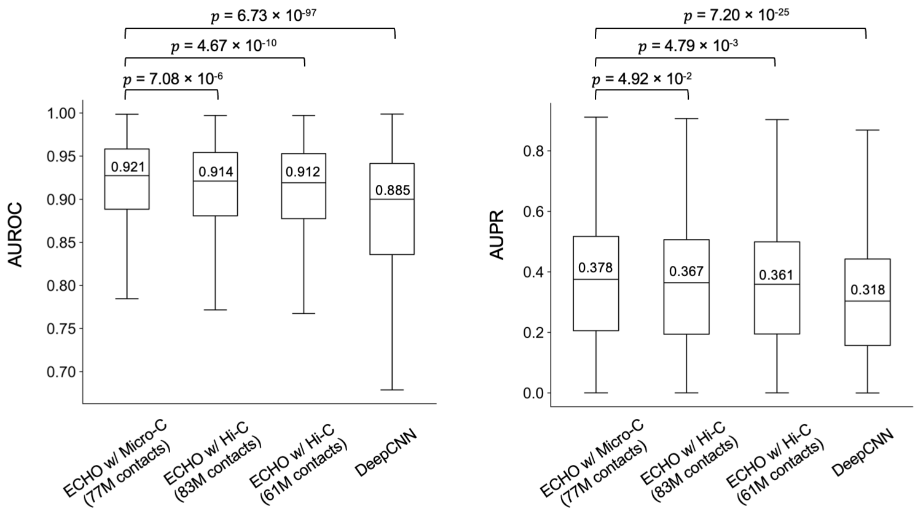

Fig R8: ECHO's performance when it is coupled with different types of contact maps. Two types of contact maps (200-bp Micro-C contact maps and up-sampled 200-bp Hi-C contact maps) are compared here. The p-values from paired t-tests indicate that Micro-C contact maps provide more precise high resolution chromatin contact information for chromatin feature prediction. The x-axis shows the contact map types and the total numbers of chromatin contacts used by ECHO (resulting from two contact value cut-off thresholds). The median AUROC and AUPR scores are provided above the median lines.

4. The results showing the enrichment of long range contacts that are attributed to CTCF motif is convincing and a reasonable validation of ECHO's ability to leverage 3D structure in imputing signal. Can a simpler CTCF loops based enrichment be also performed, using loop calls from a loop caller such as HiCCUPS or ChIA-Pet data?

> Thank you for your comments. We called chromatin loops from the Micro-C contact maps and performed the enrichment analysis as follows. If both anchors of a loop were bound by CTCF, we called it as a CTCF loop. Then we calculated the attribution scores related with CTCF binding on these CTCF loops, and plotted the attribution score distributions with respect to different ranges of contact distances (Fig R9). It is observed that the long-distance CTCF loops have higher attribution scores than short-distance CTCF loops, which indicates that the long-range chromatin contacts have greater effect in predicting CTCF binding than the short-range contacts.

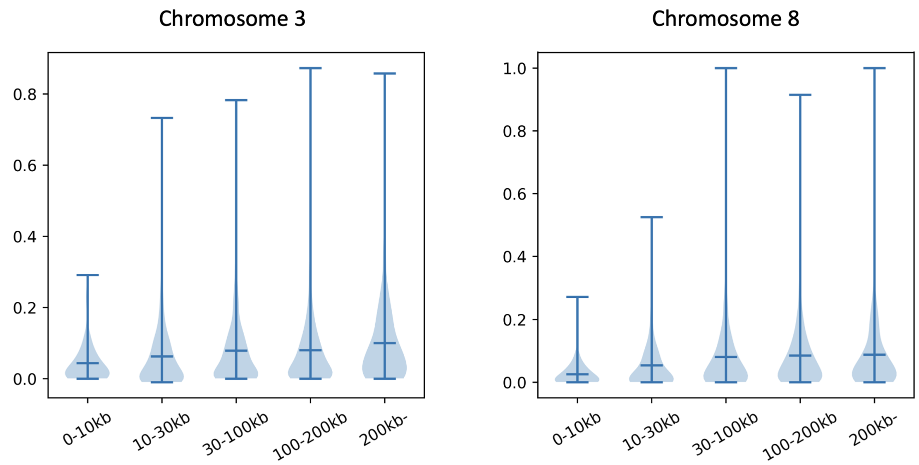

Fig R9: Violin plots of attribution scores on the called loops whose both anchors are bound by CTCF in five chromatin contact ranges (i.e., 0-10kb, 10-30kb, 30-100kb, 100-200kb, and above 200kb). The results from Chromosome 3 are on the left and the results from chromosome 8 are on the right. It is observed that the longer-range CTCF loops have higher attribution scores.

5. In line 238, authors state "In this experiment, we investigated whether specific sequence patterns in the neighborhood existed..". Are they referring to spatial or sequential neighborhood, or both?

Thank you for your suggestions. We investigated the sequence patterns mainly for spatial neighbors. To make it more clear, we have revised the sentence as follows.

"(Line 292) In this experiment, we investigated whether specific sequence patterns in the spatial neighborhood existed ..."

Minor

Authors claim "Motivated by this, most current computational models characterize TF binding and other chromatin features only from the DNA sequences". Hardly true, as ATAC/DNA-seq, and variations of ChIP-seq are also leveraged in models. I believe authors are referring to a specific subset of computational models that leverage DL - such as Basset, Basenji, Enformer and others – and aim to impute/predict signal. This needs to be clarified.

Thank you for pointing it out, we have revised this sentence as follows.

"(Line 38) Motivated by this, plenty of current deep learning based prediction models which characterize TF binding and other chromatin features such as histone marks and DNase I hypersensitive sites, only from the DNA sequences."

Paragraph 2 in intro (line 33) hardly follows the flow and is a hard transition for audiences that are not experts in this domain.

Thank you for your comments. We have added a sentence at the beginning as follows.

"(Line 44) Currently, multiple deep learning based models are proposed to predict chromatin features from DNA sequence, but most of these models predict chromatin features without utilizing the 3D chromatin organization. According to whether 3D chromatin organization is utilized, ..."

Line 39, 42: contact map concept has not been introduced or explained at all.

Thank you for your suggestions. We have added a sentence to introduce the contact maps,

"(Line 52) ... leverage the neighborhood information from 1kb resolution Hi-C contact maps which capture the spatial interactions between 1kb genomic regions..."

and a sentence as follows.

"(Line 56) In ECHO, nucleosome-resolution Micro-C contact maps which capture higher resolution chromatin contacts than Hi-C, were represented as graphs ..."

Line 46: What is a chromatin feature? It sounds like it is various properties of chromatin (Histone Mods, TF binding and chromatin accessibility) as measured by epigenomics/TF binding ChIP-seq assays and chromatin accessibility assays. Please be more specific.

Thank you for pointing it out. We have added a sentence to explain chromatin features in the introduction section.

"(Line 61) ECHO accurately predicted chromatin features including transcription factor binding, histone modifications, and DNase I hypersensitive sites, ..."

Line 218, are the candidate cis-regulatory elements referred here ccREs from the ENCODE consortium? Please clarify/cite.

Thank you for pointing it out. We have clarified that the cCREs are from ENCODE and add a citation to ENCODE.

"(Line 267) ... two candidate cis-regulatory elements (cCREs) from ENCODE [26]..."

Figure 5a, the arc between interacting regions is partially missing. Please correct.

Thank you for pointing it out. We have corrected it and made the arc bolder in Fig 5a.

References

[1] Gongcheng Hu, Xiaotao Dong, Shixin Gong, Yawei Song, Andrew P Hutchins, and Hongjie Yao. Systematic screening of ctcf binding partners identifies that bhlhe40 regulates ctcf genome-wide distribution and long-range chromatin interactions. *Nucleic acids research*, 48(17):9606–9620, 2020.

[2] Avanti Shrikumar, Katherine Tian, Žiga Avsec, Anna Shcherbina, Abhimanyu Banerjee, Mahfuza Sharmin, Surag Nair, and Anshul Kundaje. Technical note on transcription factor motif discovery from importance scores (TF-MoDISco) version 0.5. 6.5. *arXiv preprint arXiv:1811.00416*, 2018.
